# Supplementary material for: Identifying global hotspots of agricultural expansion into non-forest ecosystems
Source: Nat Commun. 2025 Nov 28;16:10739. doi: 10.1038/s41467-025-65769-x (PMC12663384; doi:10.1038/s41467-025-65769-x)
Supplement: Supplementary file 1 — Supplementary Information [file 41467_2025_65769_MOESM1_ESM.pdf]

# Supplemental Information for

## Identifying global hotspots of agricultural expansion into non-forest ecosystems

Siyi Kan [#], Jing Meng *, U. Martin Persson, Bin Chen, Samuel A. Levy, Elise Mazur, Leah Samberg, Guoqian Chen, Heran Zheng, Thomas Kastner

*Corresponding author. Email: jing.j.meng@ucl.ac.uk
# First author. Email: sykan@pku.edu.cn

**The supplementary Information file includes:**
Supplemental Tables S1
Supplemental Figures S1 to S6

## Supplemental Tables

### Table S1 Classification scheme of different land cover datasets and summary of accuracies

**(a) GlobeLand30 classification [1] (used in this study directly)**

| Class | Description |
|---|---|
| Cultivated land | It refers to the lands used for cultivating crops. Paddy fields, irrigated upland, rainfed upland, vegetable land, cultivated pasture, greenhouse land, land mainly planted with crops rarely with fruit trees or other trees, tea garden, coffee garden, and other economic cropland etc. are included in this category. |
| Forest | It refers to the lands covered with trees, the top density of which occupies over 30%. Deciduous broadleaf forest, evergreen broadleaf forest, deciduous coniferous forest, evergreen coniferous forest, mixed forest and sparse woodland the top density of which covers 10%-30% are included in this category. |
| Grassland | It refers to the lands covered by natural grass with cover density over 10%. The prairies, meadow steppes, alpine grasslands, desert steppes and lawns etc. are included in this category. |
| Shrubland | It refers to the lands covered with shrubs and the cover density over 30%. Mountain shrubs, deciduous and evergreen shrubs, and desert jungle in the desert area with the cover density over 10% are included in this category. |
| Wetland | It refers to the junction lands of land and water area, which are constantly covered by biogas or hygrophyte plants and shallow water or wet soils. Inland marsh, lake marsh, river floodplain wetland, forest/shrub wetland, peat bogs, mangrove, salt marsh etc. are included in this category. |
| Water bodies | It refers to liquid water covered region in the land area. River, lake, reservoir, pit-pond etc. are included in this category. |
| Tundra | It refers to the lands covered by lichen, moss, hardy perennial herb and shrubs in the cold and high mountain area. Shrub tundra, grass tundra, wet tundra, alpine tundra, and barren tundra, etc. are included in this category. |
| Artificial Surfaces | It refers to the surfaces formed by man-built activities. All kinds of habitation in urban and rural areas, industrial and mining area, transportation facilities etc. are included in this category. |
| Bare Land | It refers to natural covered lands with cover density lower than 10%. Desert, sand, gravel ground, bare rocks, saline and alkaline lands, etc. are included in this category. |
| Permanent snow and ice | It refers to the lands covered by permanent snow, glacier and icecap. Permanent snow, the glacier in the high mountain region, and the icecap in the polar etc. are included in this category. |

## (b) GLC_FCS30D classification [2] and related classification in this study

| GLC_FCS30D classification | | | Classification in this study |
|---|---|---|---|
| General Class | Level-1 class | Level-2 class | |
| Cropland | Rainfed cropland | Rainfed cropland | Cropland |
| | | Herbaceous cover cropland | |
| | | Tree or shrub cover cropland | |
| | Irrigated cropland | Irrigated cropland | |
| Forest | Evergreen broadleaved forest | Closed evergreen broadleaved forest | Forestland |
| | | Open evergreen broadleaved forest | |
| | Deciduous broadleaved forest | Closed deciduous broadleaved forest | |
| | | Open deciduous broadleaved forest | |
| | Evergreen needleleaved forest | Closed evergreen needleleaved forest | |
| | | Open evergreen needleleaved forest | |
| | Deciduous needleleaved forest | Closed deciduous needleleaved forest | |
| | | Open deciduous needleleaved forest | |
| | Mixed-leaf forest | Closed mixed-leaf forest | |
| | | Open mixed-leaf forest | |
| Shrubland | Shrubland | Shrubland | Shrubland |
| | | Evergreen shrubland | |
| | | Deciduous shrubland | |
| Grassland | Grassland | Grassland | Grassland |
| Tundra | Lichens and mosses | Lichens and mosses | Tundra |
| Wetland | Inland wetland | Marsh | Non-Forested Wetland |
| | | Flooded flat | |
| | | Saline | |
| | | Swamp | Forestland |
| | Coastal wetland | Mangrove | |
| | | Salt marsh | Non-Forested Wetland |
| | | Tidal flat | |
| Impervious surface | Impervious surface | Impervious surface | Impervious surface |
| Bare areas | Sparse vegetation | Sparse vegetation | Bare areas |
| | | Sparse shrubland | |
| | | Sparse herbaceous cover | |
| | Bare areas | Bare areas | |
| | | Consolidated bare areas | |
| | | Unconsolidated bare areas | |
| Water body | Water body | Water body | Water body |
| Permanent snow and ice | Permanent snow and ice | Permanent snow and ice | Permanent snow and ice |

### (c) GLCLUC classification [3] and related classification in this study

| GLCLUC classification | | | Classification in this study |
|---|---|---|---|
| **General class** | | **Subclass** | |
| Terra Firma | True desert | ≤ 7% short vegetation cover | Bare land |
| | Semi–arid | ≤ 75% short vegetation cover | Grassland and Shrubland |
| | Dense short vegetation | ≤ 100% short vegetation cover | |
| | Tree cover | < 5m trees | |
| | Tree cover | ≥ 5m trees | Forestland |
| Wetland | Salt pan | ≤ 7% short vegetation cover | Bare land |
| | Sparse vegetation | ≤ 75% short vegetation cover | Non-Forested Wetland |
| | Dense short vegetation | ≤ 100% short vegetation cover | |
| | Tree cover | < 5m trees | |
| | Tree cover | ≥ 5m trees | Forestland |
| Open surface water | | 20 - 79% of year | Non-Forested Wetland |
| Open surface water | | 80 -100% of year | Water |
| Snow/ice | | | Permanent Snow and Ice |
| Cropland | | | Cropland |
| Built–up land | | | Built–up land |
| Ocean | | | Ocean |

### (d) Summary of User's Accuracy (U.A.) and Producer's Accuracy (P.A.) for main land covers (covering approximately 95% of the total terrestrial surface)

| | | Cultivated land/ Cropland | Forestland | Grassland | Shrubland | Wetland | Bare land/ Sparse vegetation |
|---|---|---|---|---|---|---|---|
| **U.A.** | **GlobeLand30** | 82.76% | 83.58% | 72.16% | 72.64% | 74.87% | 81.76% |
| | **GLC_FCS30D** | 86.38% | 86.35% | 66.05% | 61.68% | 76.96% | 77.38% |
| | **GLCLUC** | 88.50% | 94.60% | Not available | | | |
| **P.A.** | **GlobeLand30** | Not available | | | | | |
| | **GLC_FCS30D** | 87.22% | 92.83% | 54.41% | 57.63% | 73.37% | 79.45% |
| | **GLCLUC** | 86.0% | 94.40% | Not available | | | |

**Note:** All the 3 datasets show high U.A. and P.A. for cultivated land/cropland and forestland (also for bare land in GlobeLand30 and GLC_FCS30D). Since these land covers are mapped with high accuracy, the likelihood of misclassification between them and non-forest natural land covers is reduced. As a result, the overall accuracy of non-forest natural land covers should also be high. This suggests that our estimates of total non-forest natural land conversion may have higher accuracy than those for individual non-forest land cover types.

* GLCLUC provides accuracy assessments for different time periods; we used the lowest reported accuracy for a conservative estimate. GlobeLand30 provides accuracy assessments for 2010 and GLCLUC for 2020.

# Supplemental Figures

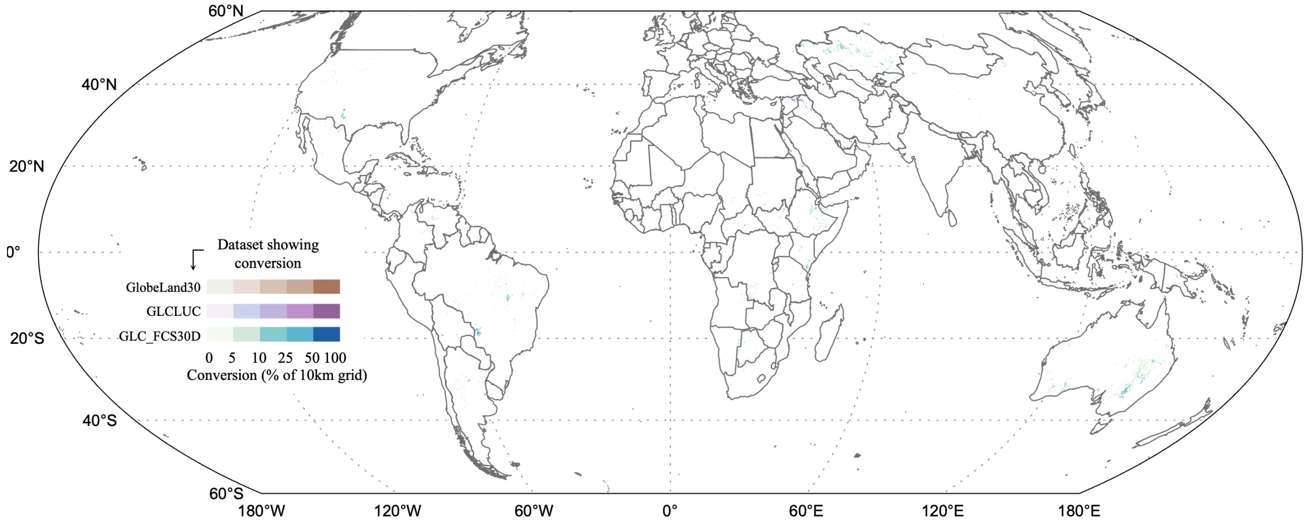


**a**

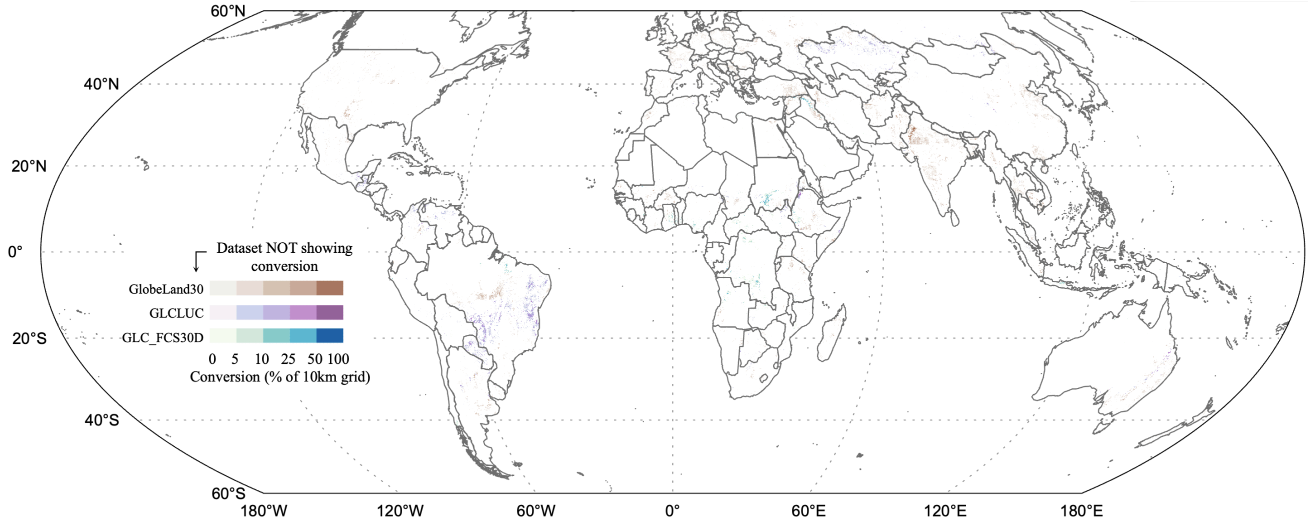


**b**

**Fig. S1 Global distribution of conversion hotspots in 2000-2020**

**a.** Distribution of pixels showing conversion in only one dataset; **b.** Distribution of pixels showing conversion in two datasets. Values are presented as percentage of 30 m by 30 m pixels in a 10 km by 10 km grid cell for which such a conversion has been identified. Only areas within the top 90% of global total conversion are colored. Administrative boundaries were modified from GADM version 4.1, aggregated and simplified for display purposes.

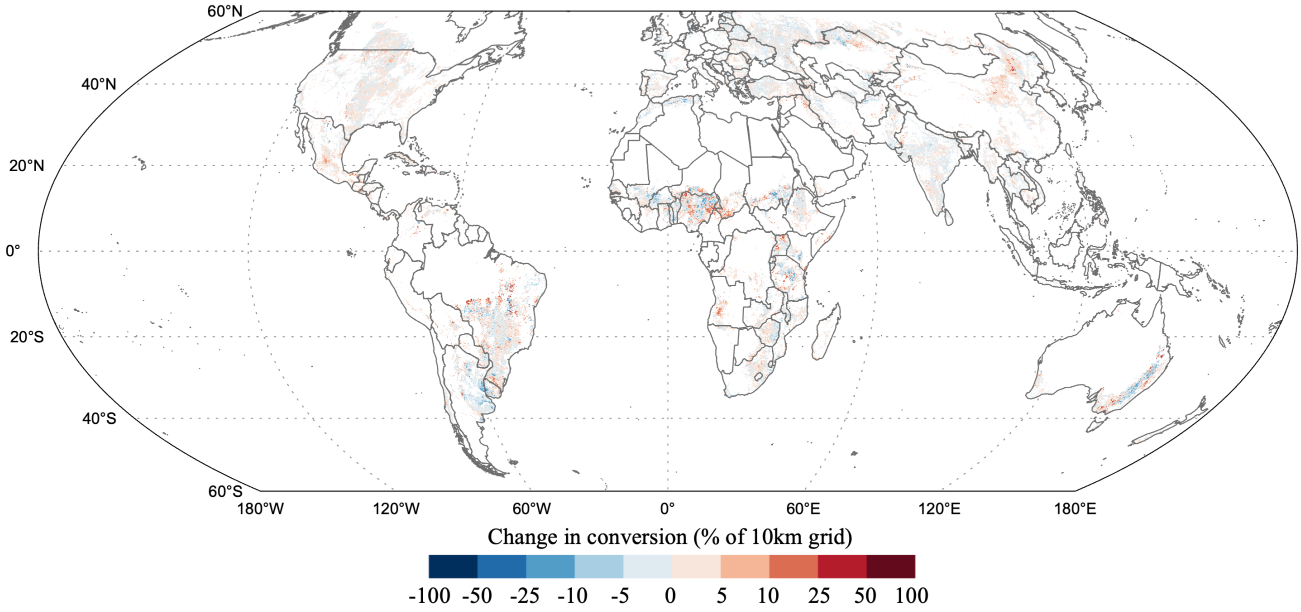


**Fig. S2 Decrease (−) and increase (+) in conversion during 2010-2020 compared to 2000-2010.** Only areas where all three datasets show conversion and their average falls within the top 90% of global total conversion are colored. Values are represented as the average change (conversion 2010-2020 minus conversion 2000-2010) estimated across the three datasets. Administrative boundaries were modified from GADM version 4.1, aggregated and simplified for display purposes.

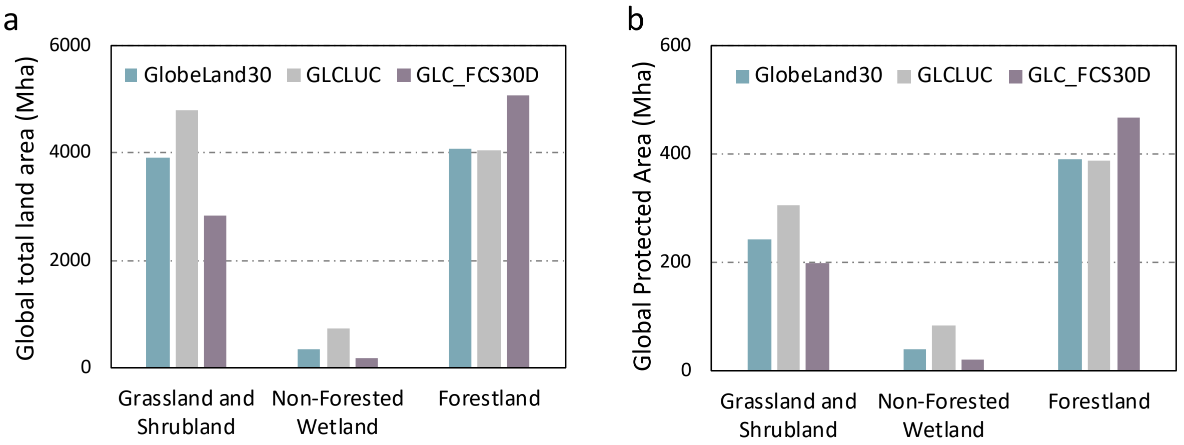


**Fig. S3 Global land area in 2000 by land cover type**

**a.** Global total land areas; **b.** Global Protected Areas (categories I-VI). By comparing Fig. S3a and Fig. S3b, it can be found that as of 2000, the proportion of forestland protected by PAs (~10%) was higher than that of non-forest land covers (~7%).

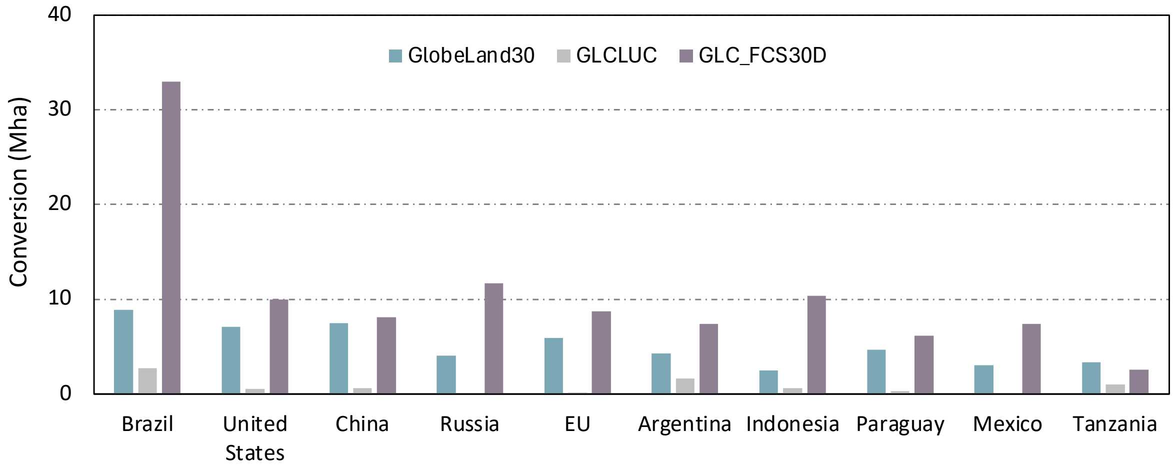


**Fig. S4 Forest conversion for top hotspot countries/regions**

2 hotspots countries/regions consistently appeared in 3 datasets and 8 hotspots countries/regions appeared in 2 datasets. The European Union (EU) is analyzed as an aggregate region.

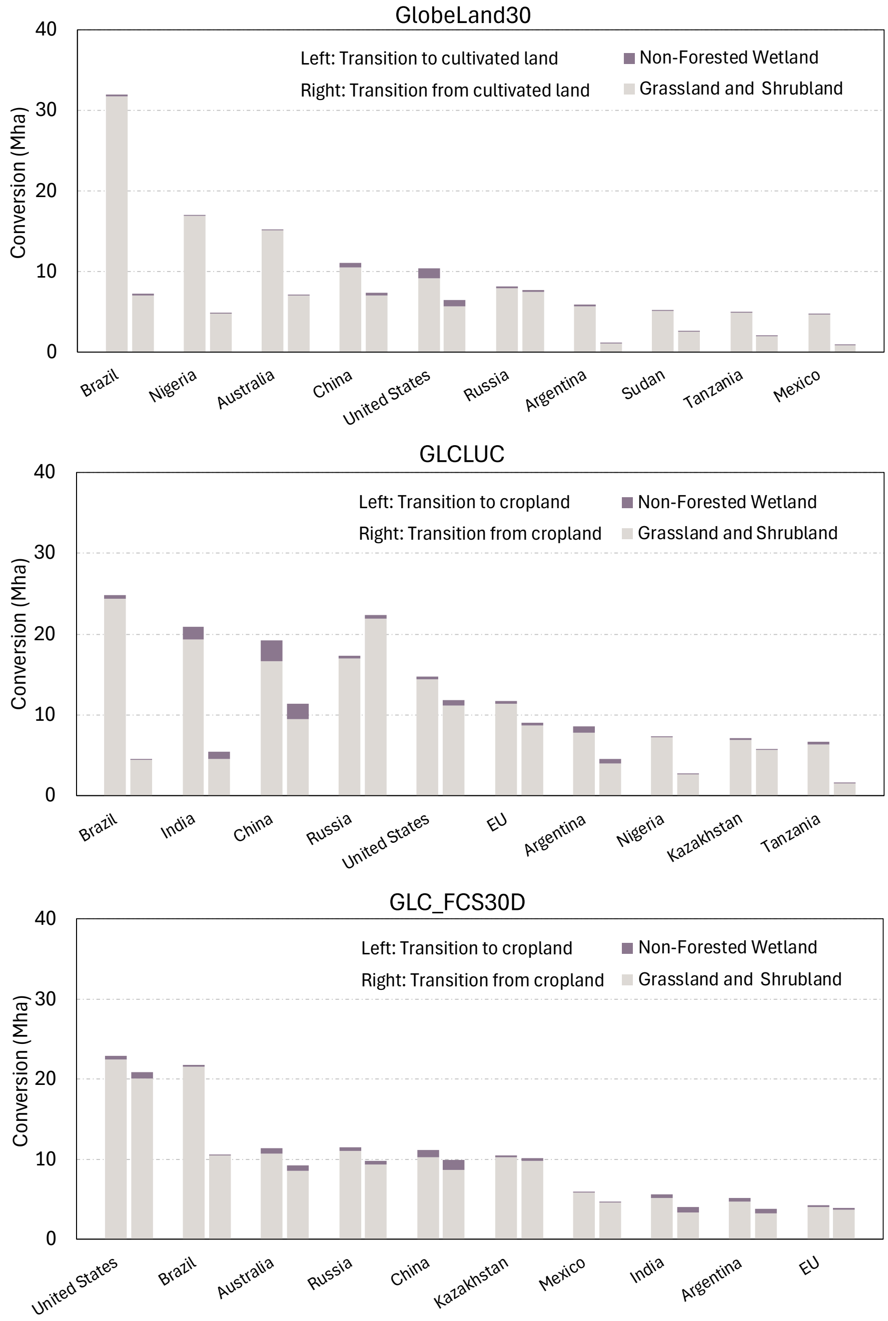


**Fig. S5 Transition between non-forest land and agricultural land in 2000-2020 for hotspot countries.** Misclassifications due to cropland fallow can occur when croplands are temporarily set aside for productivity recovery and misclassified as natural land cover. If cultivation later resumes in these areas while other croplands are set aside, this can be mistakenly recorded as conversion of natural land covers to croplands. As a result, transitions from croplands to non-forest land may reflect both real changes, such as cropland abandonment and ecological restoration, and spurious changes due to misclassification. However, distinguishing between these factors remains challenging, limiting the ability to accurately quantify spurious changes. Some cropland identification techniques help mitigate these issues. For example, GlobeLand30 identifies cultivated lands by integrating both cultivated crop / grass phenology characteristics (e.g., rotation and homogenization) and regular cropland distribution patterns (e.g., circular or rectangular fields), which can help identify fallow croplands, as they typically follow these recognizable patterns.

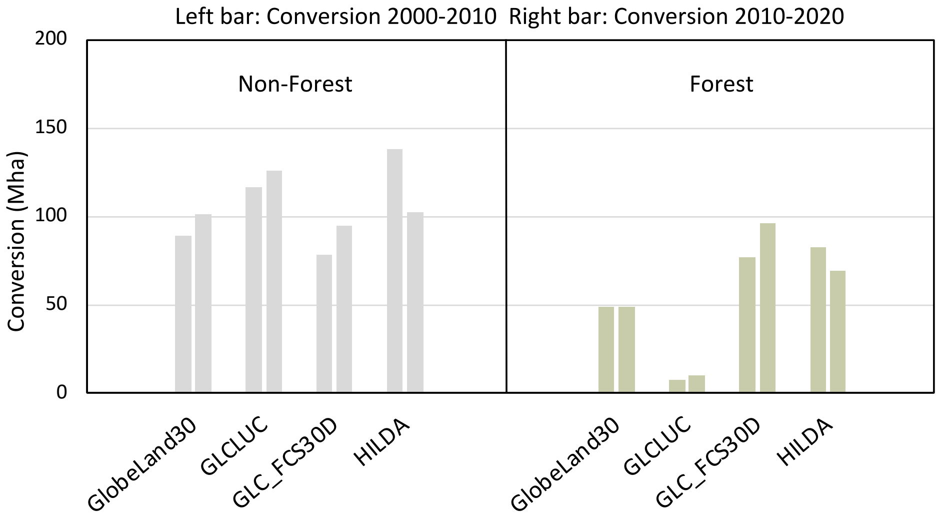


**Fig. S6 Comparison of global conversion across 4 datasets**

The HILDA dataset provided by Winkler et al.[4] differentiates cultivated pastures from croplands and unmanaged grasslands, allowing for valuable insights into the impact of not making this distinction on conversion estimates. We compared our estimates of grassland, shrubland and non-forested wetland conversion to cultivated lands/croplands with HILDA's conversion of unmanaged grass/shrubland (including wetlands) to cropland and pasture/rangeland. It is important to note that, due to the lack of high-resolution pasture distribution data (largely stemming from technological limitations), HILDA identifies pasture based on FAO's Gridded Livestock World v3 2010, which has a relatively coarse resolution of 5 arc minutes (~9.28 km at the equator) and was only available for a single year. Therefore, the results should be also interpreted with caution due to inherent uncertainties. Several other factors also contribute to the discrepancies of results, for example: (1) **Resolution differences.** The three datasets examined in this study were compiled at a 30 m resolution, while HILDA was compiled at a 1 km resolution using majority cell values, which can lead to misestimation as we do not have access to more detailed land cover fraction data. For instance, a 1 $km^2$ pixel marked as forest-to-cropland conversion may contain other land use changes within it, but we have to assume all the 1$km^2$ were converted from forest to cropland. (2) **Treatment of back-and-forth conversion.** Our estimates from the three datasets examined compared land cover 2000 with 2010 and compared 2010 with 2020 (because GlobeLand30 lacks annual data), excluding conversion to cultivated lands/croplands that were later abandoned. In contrast, our estimates based on HILDA were obtained by summing its annual transition data, capturing these back-and-forth conversions, which can affect comparability. This approach was chosen because comparing land cover changes over decadal periods at a coarse resolution may introduce greater uncertainties, as each pixel can encompass multiple land cover types. (3) **Land cover definitions**. Variations in classification criteria between datasets can also lead to differences in reported conversions, which has been elaborated in detail in the main body. (4) **Uncertainties of each land cover dataset**. All the datasets rely on different data sources and methodologies to compile land cover maps, each subject to inherent inaccuracies and uncertainties.

## Reference


1. Chen, J. *et al.* Global land cover mapping at 30m resolution: A POK-based operational approach. *ISPRS J. Photogramm. Remote Sens.* **103**, 7–27 (2015).
2. Zhang, X. *et al.* GLC_FCS30D: the first global 30m land-cover dynamics monitoring product with a fine classification system for the period from 1985 to 2022 generated using dense-time-series Landsat imagery and the continuous change-detection method. *Earth Syst. Sci. Data* **16**, 1353–1381 (2024).
3. Potapov, P. *et al.* The Global 2000-2020 Land Cover and Land Use Change Dataset Derived From the Landsat Archive: First Results. *Front. Remote Sens.* **3**, (2022).
4. Winkler, K., Fuchs, R., Rounsevell, M. & Herold, M. Global land use changes are four times greater than previously estimated. *Nat. Commun.* **12**, 2501 (2021).
